# A simple management option for chronically impacted sharp tracheobronchial foreign bodies in children

**DOI:** 10.1186/s40463-018-0272-0

**Published:** 2018-04-10

**Authors:** Sherif Idris, Russell A. Murphy, Manisha Witmans, Hamdy El-Hakim

**Affiliations:** 1 Pediatric Otolaryngology, Divisions of Otolaryngology Head & Neck Surgery and Pediatric Surgery, Department of Surgery, The Stollery Children’s Hospital & University of Alberta Hospitals, 2C3.57 Walter MacKenzie Centre, Edmonton, Alberta T6G 2R7 Canada; 2 Division of Otolaryngology – Head and Neck Surgery, University of Saskatchewan, Saskatoon, Saskatchewan Canada; 3 Division of Pediatric Pulmonology, Department of Pediatrics, The Stollery Children’s Hospital & University of Alberta Hospitals, Edmonton, Alberta Canada

## Abstract

Distally impacted chronic tracheobronchial sharp foreign bodies in children are a management challenge that presents with clinical subtlety and extreme variability. The use of image guided techniques, imaginative instrumentation, tracheotomy, thoracotomy, and even extracorporeal membrane oxygneation have been reported. Endoscopy is made difficult by the distal location, inflammatory reaction with granulation tissue formation, and bleeding obscuring the foreign body. Our aim is to describe our experience with two children who had removal of aspirated impacted sharp metallic foreign bodies from the distal airway using rigid bronchoscopy, preceded by maximal medical therapy.

## Background

Aspirated foreign bodies most commonly occur in children younger than 3 years of age, and may relate to immature dentition and a poorly coordinated swallowing mechanism [1, 2]. The increased work of breathing and higher respiratory rates characteristic of this group is also thought to predispose them to aspiration [3]. This population incurs about 75% of airway foreign bodies; with organic foreign bodies, such as peanuts and other seeds, being the commonest agents [4]. In the mean time, inorganic foreign bodies, including plastic, wooden, and sharp metal object aspirations are more common in older children [5]. Sharp foreign body aspiration are particularly dangerous due to the increased likelihood of life threatening complications including pneumothorax, bronchial rupture, and pericardial tamponade [6]. Several large series from Muslim nations where headscarf pin aspiration is an increasing public health concern has recently demonstrated these hazards [6–9].

Tracheobronchial foreign bodies (TFBs) can manifest as life-threatening emergencies requiring immediate intervention or, in some cases, may go unnoticed for weeks or even months [1]. Undiagnosed airway foreign bodies can lead to mechanical respiratory compromise and/or chemical pneumonitis, and may mimic/present as chronic pulmonary infections, bronchiectasis, asthma, lung collapse, or lung abscess [10]. It is thought that the longer the foreign body remains, the higher the likelihood of distal migration in the tracheobronchial tree and that of developing significant airway edema, granulation tissue, and infection [4]. Late-presenting TFBs are usually impacted in the bronchial lumen, making removal particularly challenging. Extraction via bronchoscopy may not be possible, necessitating alternative techniques and potentially open surgical procedures [11]. These may include any combination of the use of intraoperative fluoroscopy complimented with rigid and flexible endoscopy [12], tracheotomy with postural percussion [13], and balloon dilation of the airway to facilitate foreign body extraction [14]. Open surgical techniques used included bronchotomy and pulmonary resection. However, little is reported in the literature about the use of medical management to optimize the airway in the presence of these complicating factors. The aim of this report is to describe the diagnosis and management of two children with sharp metallic foreign body (FB) impaction of the distal airway that were treated with medical management which facilitated endoscopic extraction.

## Reports

### Case 1

**Fig. 1:**
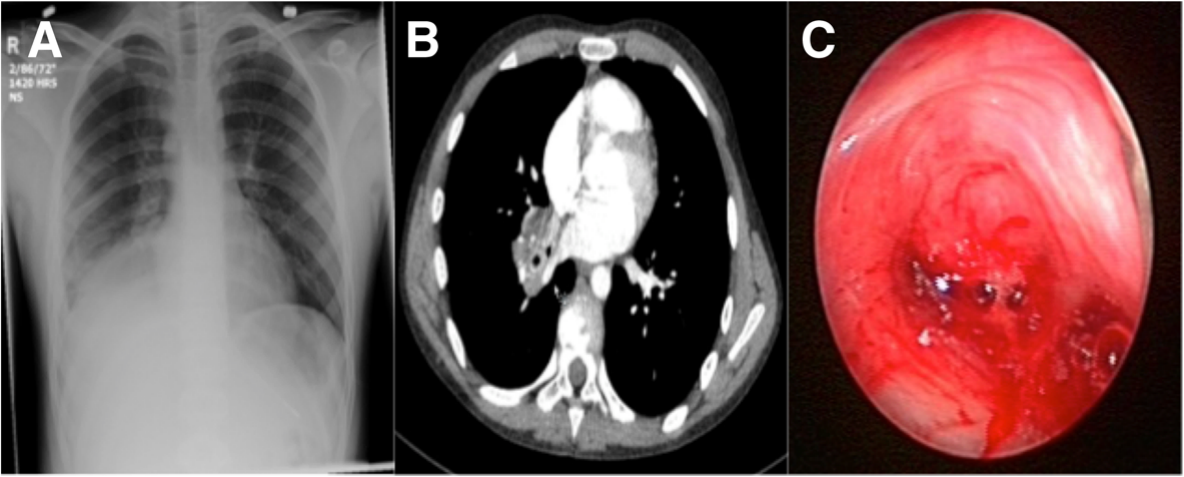
Case 1 - Posteroanterior (PA) chest x-ray showing complete right lower lobe opacification (**a**). CT of the chest showing right perihilar parenchymal opacification with partial collapse of the right lobe bronchus (**b**). Endoscopic view of extensive granulation tissue in the right main stem bronchus and excessive bleeding (**c**)

A 13-year-old boy presented with a 2-month history of hemoptysis, productive cough, and shortness of breath and was referred to the pediatric pulmonology service. He had a family history of tuberculosis and was being investigated for necrotizing pneumonia. He was previously diagnosed with pneumonia and was treated with antibiotics, but returned to the hospital because of worsening respiratory distress. His initial chest x-ray showed complete opacification of the right lower lobe suggestive of consolidation (Fig. 1a). Computed tomography (CT) imaging of the chest was performed the following day and showed dense consolidation in the right lower lobe with evidence of hemorrhage into the right basal segmental bronchi, suggestive of a necrotizing pneumonia (Fig. 1b). Repeat chest x-rays over the following days showed worsening consolidation of the right lower lobe with development of a right pleural effusion and abscess formation. He was taken to the operating room for a flexible fiberoptic bronchoscopy and bronchoalveolar lavage by the Pediatric Pulmonology service. Copious purulent secretions and granulation tissue was noted arising from the right main stem bronchus. The Pediatric Otolaryngology service was consulted, and a rigid bronchoscopy was performed. Subsequent instrumentation resulted in significant bleeding that was controlled with topical 1:1,000 epinephrine solution. A metallic foreign body was identified in the bronchus intermedius but was impossible to retrieve due to bleeding (Fig. 1c). The procedure was aborted and the patient was managed medically with intravenous dexamethasone (0.5 mg/kg body weight/dose, every 8 h), ceftriaxone (1 g daily), clindamycin (600 mg every 8 h), and nebulized budesonide (500 μg every 6 h) for 48 h before a repeat bronchoscopy was attempted. He was then taken back to the operating room and flexible fiberoptic bronchoscopy revealed a significant reduction the amount of granulation tissue and purulent discharge in the right main stem bronchus. A rigid bronchoscopy was then performed with a size 6 adolescent ventilating bronchoscope and a 0° telescope (2.7 mm × 40 cm). The foreign body was identified in the bronchus intermedius and was easily removed with telescopic alligator optical forceps. Minimal bleeding at that site stopped spontaneously.

### Case 2

**Fig. 2:**
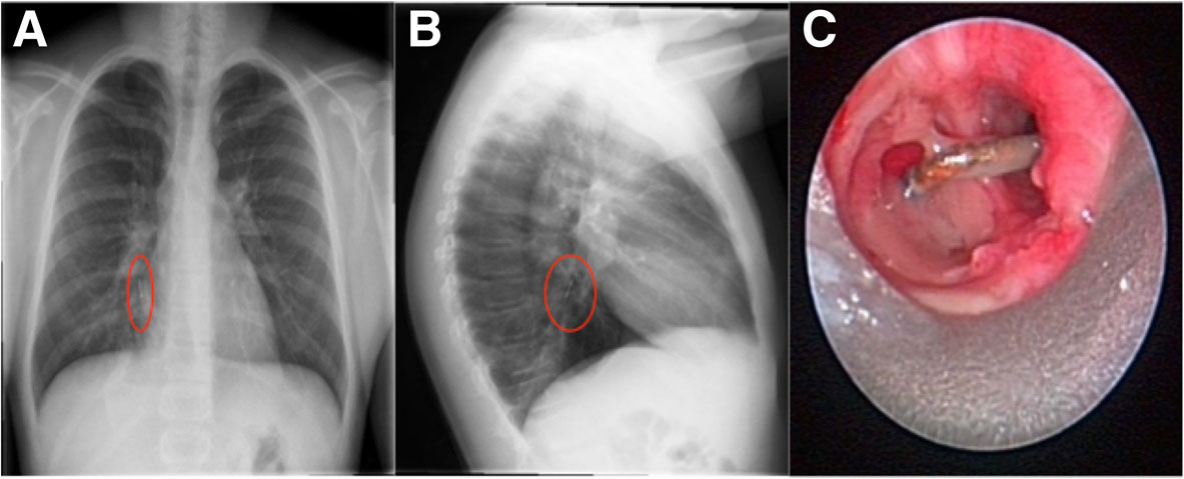
Case 2 - Posteroanterior (PA) chest x-ray showing metallic foreign body in right lower lobe bronchus (**a**). Lateral chest x-ray shows a metallic foreign body and surrounding consolidation (**b**). Endoscopic view of an impacted metallic foreign body with surrounding granulation tissue and inflammation (**c**)

13-year-old boy, previously healthy, presented with a 4-month history of hemoptysis, shortness of breath, and right chest pain. His chest x-ray showed a vertically oriented 1.4 cm metallic foreign body in the right lower lobe bronchus and a region of focal airspace consolidation at the posterior basal segment of the right lower lobe suggestive of pneumonia (Fig. 2a and b). He was taken to the operating room for a flexible fiberoptic bronchoscopy by the Pediatric Pulmonary service. Copious purulent secretions and granulation tissue was noted in the right lower lobe bronchus. A metal nail was visualized embedded in the right lower lobe bronchus and appeared to be partially encased in granulation tissue. The Pediatric Otolaryngology service was consulted to perform a rigid bronchoscopy. Attempts were made to remove the nail but were unsuccessful due to the surrounding granulation tissue and inflammation (Fig. 2c). Significant bleeding resulted with further attempts and the procedure was aborted. The patient was then placed on a 72-h course of IV dexamethasone (0.5 mg/kg body weight/dose, every 8 h), levofloxacin (10 mg/kg body weight/dose daily), and nebulized budesonide (500 μg every 6 h) before a repeat bronchoscopy was attempted. After completion of the steroids and antibiotics he was taken back to the operating room and rigid bronchoscopy was then performed with a size 6 adolescent ventilating bronchoscope and a 0° telescope (2.7 mm × 40 cm). The amount of granulation tissue and surrounding airway inflammation had significantly improved compared to prior to medical management. The metallic foreign body was identified in the right lower lobe bronchus was easily removed with telescopic optical forceps (Fig. 2c).

## Discussion

Tracheobronchial foreign body (TFB) aspiration is an important differential consideration in a child presenting with hemoptysis. Although the majority of patients with TFB aspiration are under 3 years of age, it may also occur in older children and adolescents. However, in the older age group the incidence of inorganic foreign body aspiration is greater [11]. During temporary placement of a needle between the lips, for example, accidental aspiration may occur, as had been reported among young (median age 15 years) Muslim girls who wore headscarves. Kaptanoglu et al. [6] showed that sharp pins were responsible for the majority (*n* = 11/13) of the complications encountered, including bronchial rupture, peripheral migration, and pneumothorax. Eight percent of these patients required multiple bronchoscopies or thoracic surgical intervention for treatment of the associated morbidity and/or to remove the foreign body. Repeat bronchoscopic intervention is far from benign, and previous unsuccessful attempts at bronchoscopic removal have been shown to increase thoracotomy rates up to 27% in series including sharp and metallic objects [15].

Although they manifest with a much less dramatic picture than anticipated, late presentation of chronic TFB still accounts for up to 48% of the total [16]. Delayed diagnosis further complicates retrieval of the TFB as prolonged impaction increases the risk of infection, erosion, and granulation tissue formation [17, 18]. Martin et al. [4] found that 89% (*n* = 31/35) of delayed presentations (defined as ≥14 days from the witnessed time of foreign body aspiration or from time since onset of symptoms) were associated with intraluminal granulation tissue on endoscopy and 46% of all patients were reported to have granulation tissue obstructing more than 50% of the involved airway lumen (which they defined as significant). This study also demonstrated that 60% (*n* = 21/35) of patients had pus in the involved airway. These features can lead to failure of endoscopic extraction due to limited visualization and bleeding, thus requiring open surgical procedures such as bronchotomy and pulmonary resection. Major complications of such procedures include empyema and bronchopleural fistula. Nine of these patients developed pulmonary complications later on. Specifically, five children required a long hospital admission secondary the development of pneumonia; another four developed bronchiectasis (confirmed on CT); and two continued to manifest with chronic pulmonary symptoms. At least one repeat laryngobronchoscopic examination was performed in 26% (*n* = 9/35) of the patients due to incomplete removal of the TFB on first procedure (4 patients), granulation tissue at the level of subglottis (2 patients), exposed cartilage (1 patient), and unclear reasons (2 patients). No patients in their study required lobectomy, however one child required an open approach for a migrated foreign body and another had presented with a previously treated bronchopleural fistula via a thoracotomy approach for a second foreign body at same time of aspiration. They reported a 26% (*n* = 9/35) overall risk for developing respiratory complications after removal of a long-standing aspirated TFB. The main short-term complication was pneumonia and the main long-term complication was bronchiectasis. This high complication rate is a distinct feature of children with chronic TFB aspiration and should be taken into consideration when counseling the patient’s family and managing expectations. Patients with chronic TFBs were also found to have a mean hospital stay of 2.5 times longer than patients with acute presentations. The extended hospital stay may be due to pneumonia, need for a repeat procedure, or further respiratory investigations for suspected chronic complications.

In our cases we administered high-dose systemic and inhaled steroid for 48–72 h prior to repeating laryngobronchosocpy. This reduced the extent and vascularity of the granulation tissue. We also administered systemic intravenous antibiotics over the same time period, which may have decreased the burden of underlying infection in the involved airways. This may have enhanced the effect of the steroids and facilitated the safe removal of the foreign body. We think that the consideration of ameliorative medical therapy should be given to those patients who are clinically stable. In this patient population, this would avoid prolonged and repeated general anesthesia with potential deterioration. Ameliorative medical therapy may also provide an easier and more cost-effective alternative. Although our study was limited to inorganic, metallic TFBs, decreasing the impact of granulation and secondary infection using ameliorative medical therapy may also be considered in patients with organic TFB aspiration, as previous reports showed similar association with granulation, pus, or respiratory complications to inorganic foreign bodies [4].

## Conclusion

In distally impacted sharp TFB the soft tissue reaction encountered during the first bronchoscopy may prohibit successful extraction. Treatment for 2–3 days with high dose inhaled and systemic steroids and systemic antibiotics may negate recourse to prolonged and repeated endoscopies and aggressive surgical methods.

## Abbreviations

TFBs: Tracheobronchial foreign bodies
TFB: Tracheobronchial foreign body
FB: Foreign body
CT: Computed tomography
PA: Posteroanterior
